# Carbohydrate Antigen (CA 19-9) Surge: Unraveling the Enigma of Elevated Levels in the Setting of Benign Etiologies

**DOI:** 10.7759/cureus.57469

**Published:** 2024-04-02

**Authors:** Nida Ansari, Sacide S Ozgur, Damian Besada, Noor Bittar, Gabriel Melki, Kanthi Badipatla, Derick Christian, Yana Cavanagh

**Affiliations:** 1 Internal Medicine, St. Joseph’s Regional Medical Center, Paterson, USA; 2 Internal Medicine, St. Joseph's Regional Medical Center, Paterson, USA; 3 Internal Medicine, St. George's University School of Medicine, Great River, USA; 4 Gastroenterology, St. Joseph's Regional Medical Center, Paterson, USA; 5 Surgery, St. Joseph's Regional Medical Center, Paterson, USA

**Keywords:** pancreas disease, magnatic resonance cholangiopancreaticography(mrcp), bilirubin, ca-19-9, endoscopy ercp

## Abstract

Carbohydrate antigen 19-9 (CA 19-9) is widely recognized as a tumor marker primarily associated with pancreatic cancer. However, its elevation in benign pancreaticobiliary conditions complicates its diagnostic utility. We present the case of a 39-year-old male with no significant medical history who presented with symptoms of abdominal pain, nausea, vomiting, and diarrhea. The initial diagnosis suggested viral enteritis, but the subsequent worsening of symptoms led to further investigation. Elevated white blood cell counts, bilirubin levels, and liver function tests prompted magnetic resonance cholangiopancreatography (MRCP), which revealed dilated bile ducts and acute cholecystitis. Following endoscopic retrograde cholangiopancreatography (ERCP), significant hemobilia was observed, raising suspicions of cholangiocarcinoma. Despite extensive investigations, including CT angiography, MRCP, and repeat ERCPs, no malignancy was detected. Remarkably, the CA 19-9 level was elevated to 904 U/mL after the initial ERCP and uptrended to 7380 U/mL. These levels, however, normalized to 13 U/mL within two weeks of discharge. While CA 19-9 is a valuable marker in the diagnosis of pancreatic cancer, its elevation in benign pancreaticobiliary conditions necessitates cautious interpretation. In our case, choledocolithasis, cholangitis, and biliary manipulation appeared to have contributed to a transiently elevated CA 19-9. Clinicians must consider the entire clinical context when evaluating elevated CA 19-9 levels to avoid misdiagnosis and ensure appropriate patient management.

## Introduction

CA 19-9 (carbohydrate antigen 19-9, cancer antigen 19-9, or siaylate lewis-a antigen) is a biochemical tumor marker associated with pancreatic pathologies [1]. It was first noted in 1979 using a mouse monoclonal antibody in a colorectal carcinoma cell line [1]. CA 19-9 is the most commonly associated tumor marker with pancreatic cancer [1]. While this is useful in the diagnosis of pancreatic cancer, CA 19-9 has been known to be elevated in a wide variety of pancreaticobiliary etiologies [1]. The clinician needs to be able to discern whether CA 19-9 is elevated in the setting of malignancy or whether its elevation is the result of overexpression in the setting of a benign disease. Here, we present a case of elevated CA 19-9 in the setting of choledocolithasis and cholangitis, managed with ERCP.

## Case presentation

A 39-year-old male with no significant past medical history presents to the emergency department (ED) with right upper quadrant abdominal pain, nausea, vomiting, and diarrhea for two weeks. He also reported fever and chills that have been ongoing for the past two weeks and excessive fatigue for the past month. Vitals showed a temperature of 37.0°C, a heart rate of 77 per minute, a respiratory rate of 18 breaths per minute, a blood pressure of 123/84 mmHg, and an oxygen saturation of 99% on room air. Labs revealed an elevated white blood cell count (WBC) of 16.5 x 109 U/L, elevated aspartate aminotransferase (AST) of 209 U/L, elevated alanine aminotransferase (ALT) of 147 U/L, alkaline phosphatase (Alk Phos) of 209 U/L, elevated total bilirubin of 6.6 mg/dL, and elevated direct bilirubin of 4.50 mg/dL (Table 1).

**Table 1:** Initial lab values are seen in the ED along with the reference range.

Lab Value	Initial Value in ED	Reference Range
White blood cell count (WBC)	16.5 x 10^3^ U/L	4.5 to 11.0 x 10^3^ U/L
Aspartate aminotransferase (AST)	209 U/L	13 to 39 U/L
Alanine aminotransferase (ALT)	147 U/L	7 to 52 U/L
Alkaline phosphatase (Alk Phos)	209 U/L	34 to 104 U/L
Total bilirubin	6.6 mg/dL	0.3 to 1.1 mg/dL
Direct bilirubin	4.50 mg/dL	0.00 to 0.20 mg/dL

**Figure 1:**
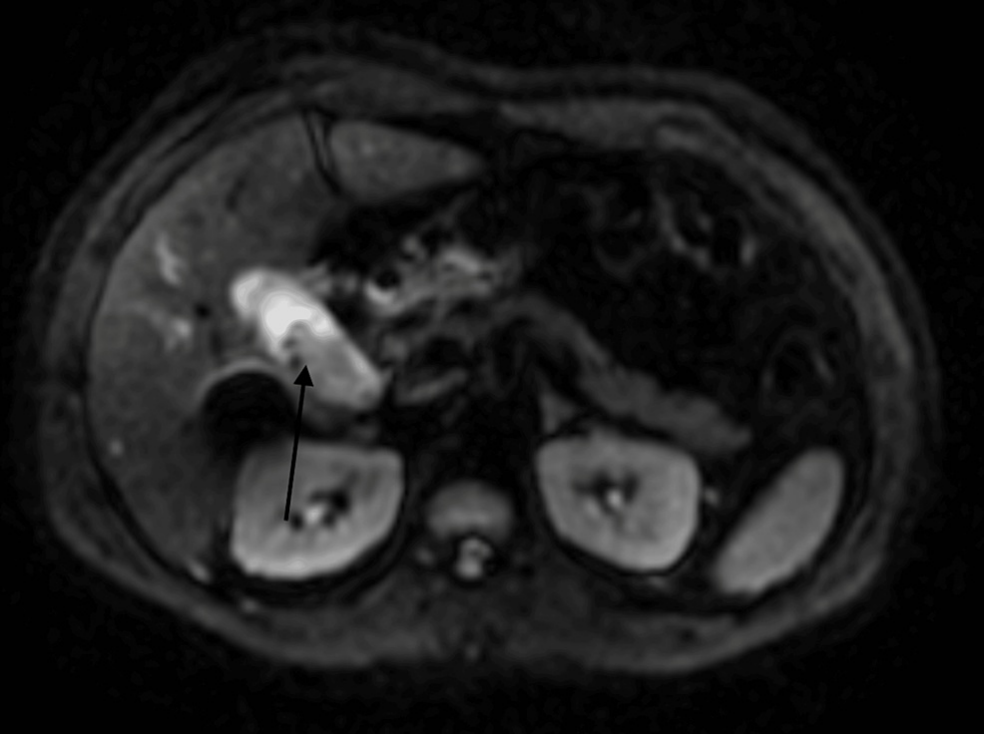
An axial view of magnetic resonance cholangiopancreatography shows cholecystitis and dilated intrahepatic and extrahepatic ducts, more prominent in the left hepatic lobe.

Due to suspicion of choledocholithiasis, a magnetic resonance cholangiopancreatography (MRCP) without contrast was performed, which revealed cholecystitis and dilated intrahepatic and extrahepatic ducts, more prominent in the left hepatic lobe (Figure 1). With these findings, the decision was made to perform endoscopic retrograde cholangiopancreatography (ERCP) the following day. 

**Figure 2:**
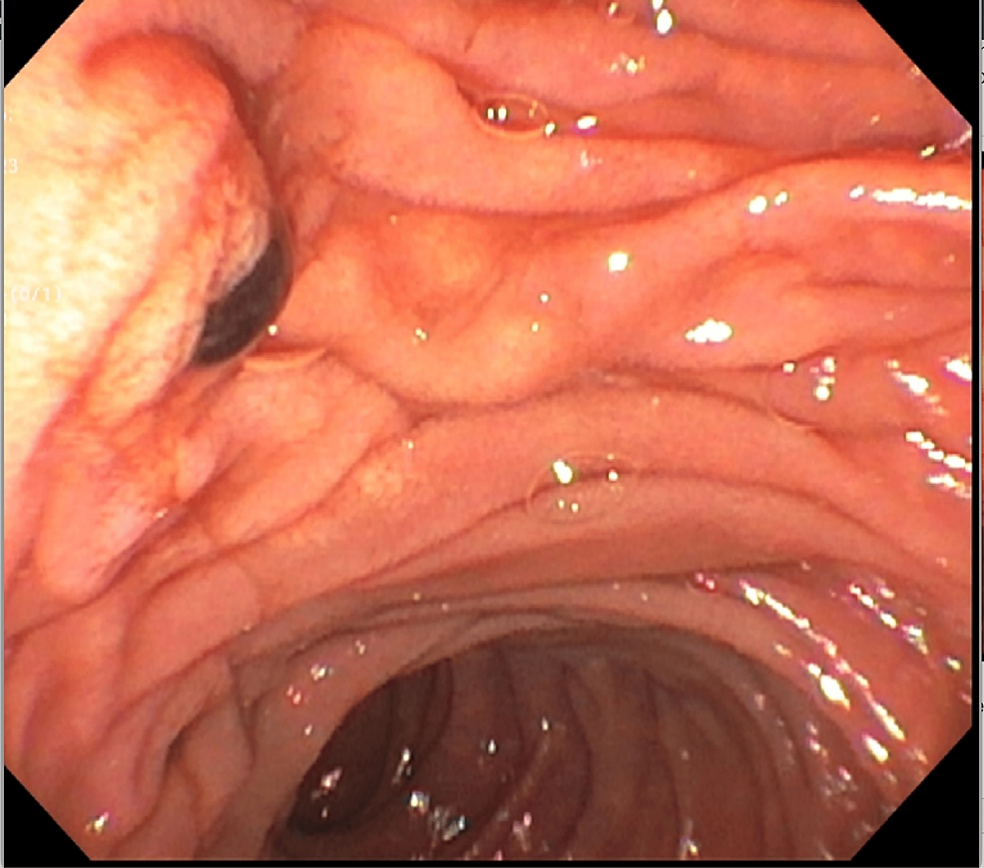
On ERCP, a moderate number of clots were seen exiting through the major papilla's orifice.

At the time of ERCP, a moderate number of clots were seen exiting through the major papilla's orifice (Figure 2). The middle and upper thirds of the main bile duct contained filling defects thought to be intraductal clotting. A biliary sphincterotomy was made, and the biliary tree was swept, discovering clotted blood products. A plastic stent with one internal flap was placed into the CBD. The significant spontaneous hemobilia identified on the ERCP raised suspicion of cholangiocarcinoma. 

**Figure 3:**
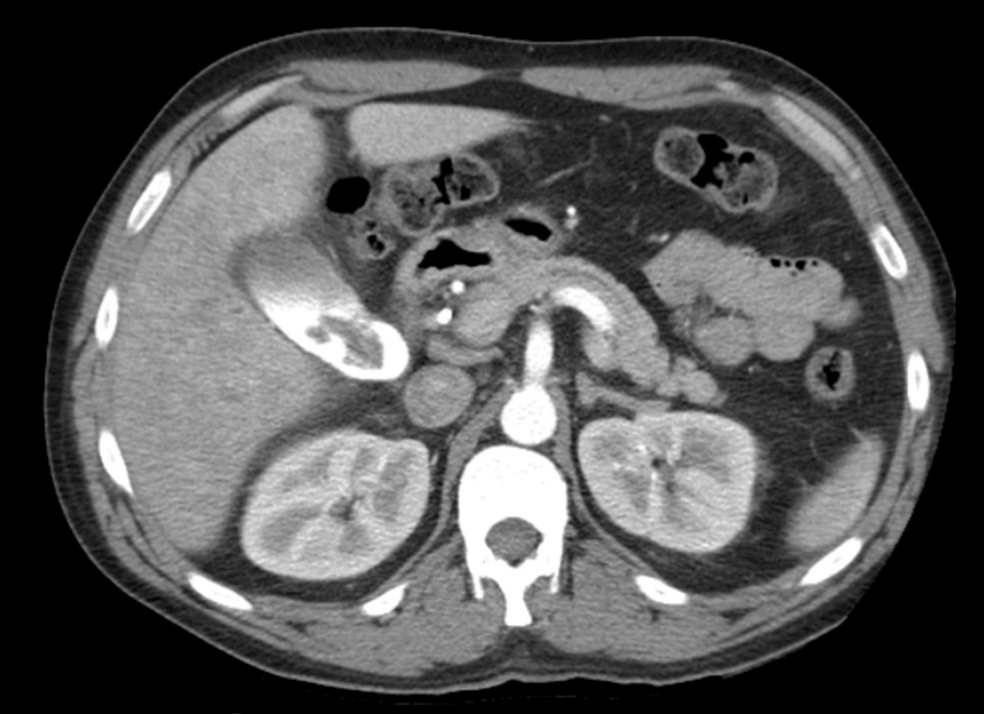
Axial view of CT angiography reveals an irregular filling defect in the gallbladder lumen, consistent with hemorrhage.

An abdominal computed tomography with angiography (CTA) was performed and revealed an irregular filling defect in the gallbladder lumen, consistent with hemorrhage (Figure 3). Blood work was ordered to investigate further etiologies of hemobilia, including carcinoembryonic antigen (CEA), CA 19-9, and IgG4. Only CA 19-9 was significant at 904 U/mL.

**Figure 4:**
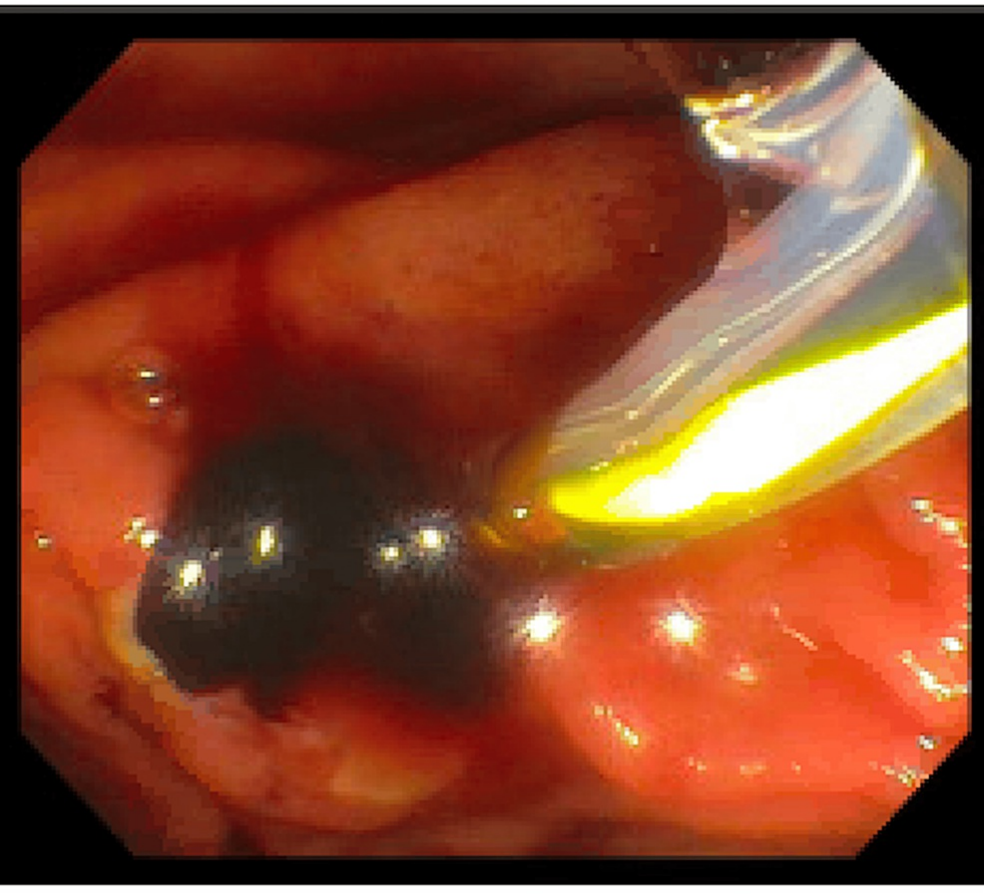
Upon sweeping the biliary tree, blood and bile were found.

**Figure 5:**
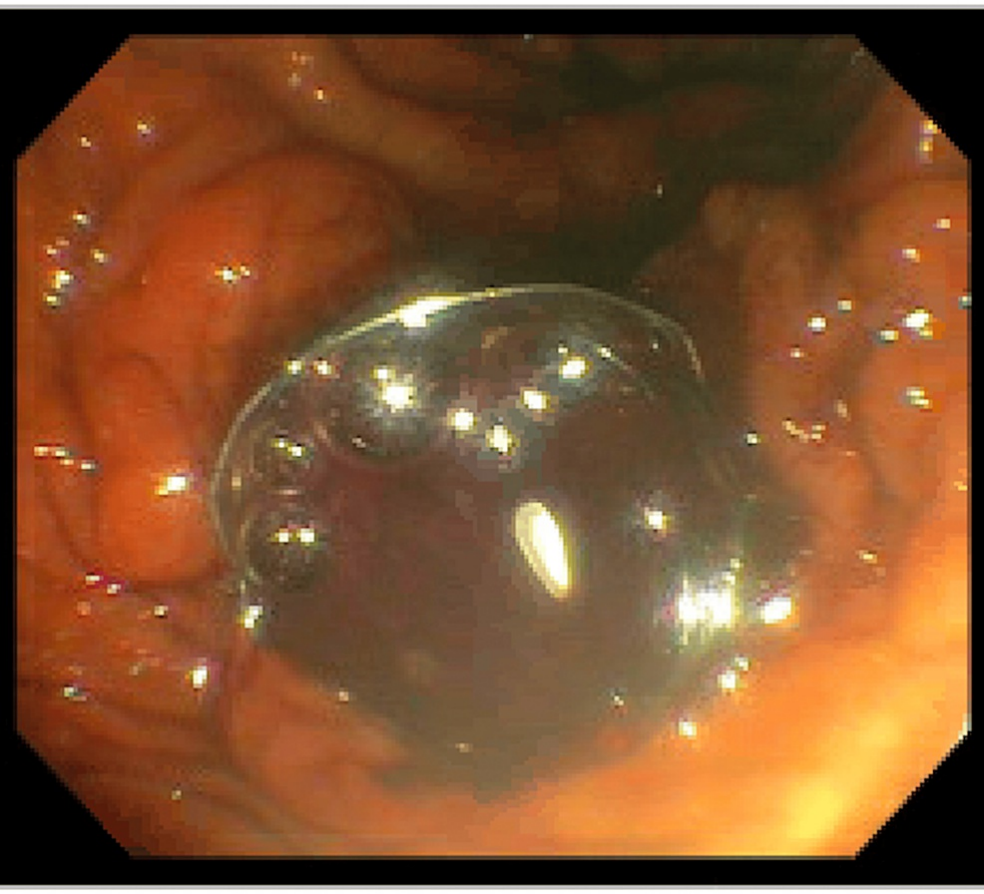
A metal stent was placed in the common bile duct to facilitate continued patency and provide radial pressure to tamponade potential ductal bleeding.

The patient had a rise in bilirubin for two consecutive days, and at that time, the decision was made to contact advanced endoscopy. A repeat ERCP was performed, and the previous stent was removed due to the concern for obstruction. The left intrahepatic ductal system was diffusely dilated. Clots with dark bile were found and swept from the duct (Figure 4). A metal stent was placed in the common bile duct to facilitate continued patency and provide radial pressure to tamponade potential ductal bleeding (Figure 5).

As there was a concern for active GI bleeding, interventional radiology was consulted. A CT angiogram of the abdomen and pelvis was performed with no persistent extravasation of contrast into the biliary tree to suggest active bleeding. Given the patient's elevated CA 19-9 and LFTs, the patient had a third ERCP with endoscopic ultrasound (EUS) with SpyGlass direct cholangioscopy.

**Figure 6:**
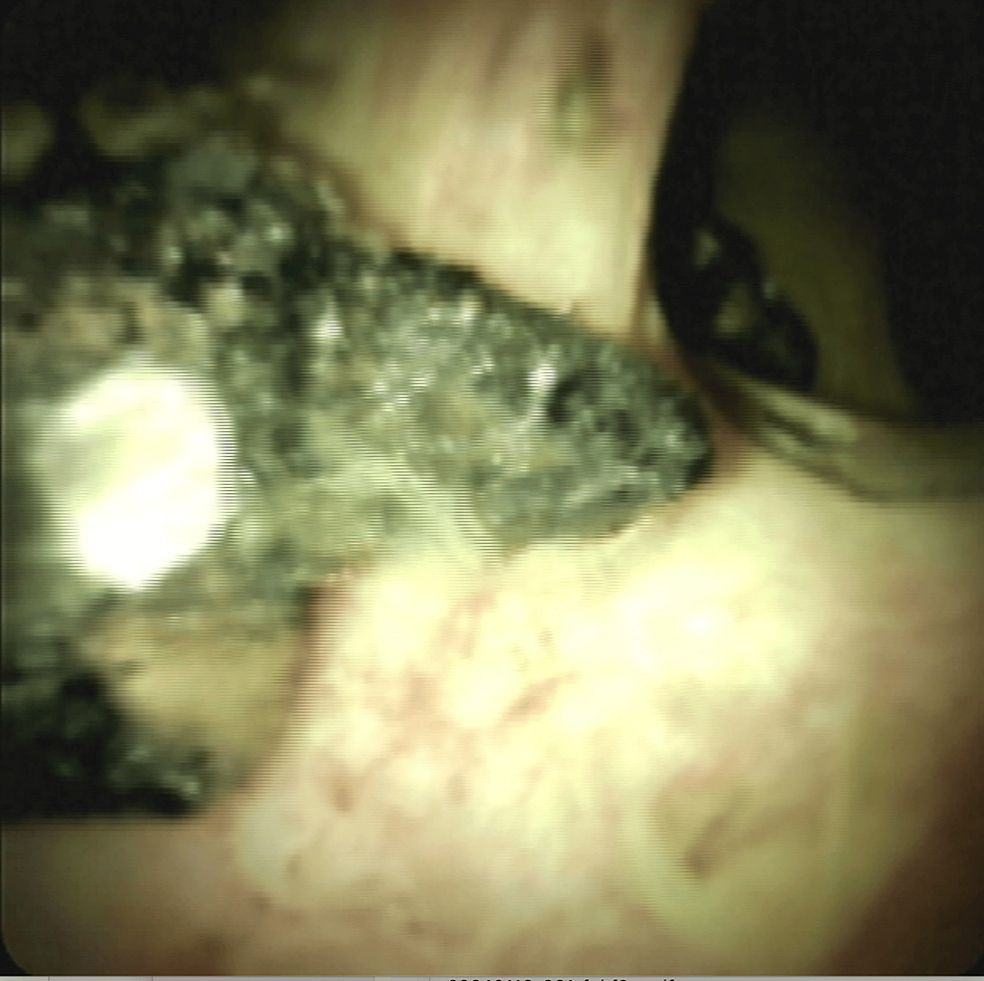
Stone found in the bile duct while utilizing SpyGlass.

EUS did not reveal significant pancreas pathology, and dilated bile ducts were visualized in the liver. One stent was visualized in the CBD. During the ERCP, hemobilia was found again in the biliary tree. The bile ducts were examined using SpyGlass, which revealed two stones in the left main and common hepatic duct, with the largest measuring 4 mm in diameter (Figure 6). Electrohydraulic lithotripsy was successfully performed for the intrahepatic stones. The metal stent that was previously placed was removed, and the bile ducts were evaluated with SpyScope. Diffuse biliary epithelial irregularities were noted throughout. Biopsies were taken with SpyBite in the right main hepatic duct and right intrahepatic branches. A new plastic stent with a single external and internal flap was placed in the CBD. Biopsies revealed benign tissue with very mild chronic inflammation and reactive fibrosis.﻿

A follow-up MRCP was done three days later, which showed severe intrahepatic and extrahepatic biliary dilatation with abrupt caliber change at the papilla, with CBD size noted to be 2.1 cm. Cholecystolithiasis was also found. CA 19-9 was repeated and was found to markedly increase to 7380 U/mL. Given the patient's hemobilia, persistently elevated total bilirubin, and elevated CA 19-9, a repeat ERCP was done to obtain additional biopsies of the biliary system to rule out malignancy. ERCP revealed one plastic stent emerging from the major papilla, which was removed. The biliary tree was found to be diffusely dilated. Upon lavage of the biliary tree, sludge, two stones, debris, clots, and pus were swept from the duct. A metal stent was placed in the CBD. The following day, the total bilirubin was found to be 3.2 mg/dL, markedly reduced from 9.8 mg/dL the day of the last ERCP. 

The patient's clinical status improved, and he was discharged with outpatient follow-up with gastroenterology, oncology, and surgery for outpatient laparoscopic cholecystectomy. CA 19-9 was repeated two weeks after discharge and was found to be 13 U/mL (Table 2). Upon evaluation with oncology, it was concluded that the elevation of CA 19-9 was secondary to a benign etiology.

**Table 2:** Trend of total and direct bilirubin and CA 19-9 in a correlation of ERCPs during and after the patient's hospital stay.

Hospital Day	1	2	3	4	5	6	7	8	9	10	11	12	13	14	15	Two weeks Later
ERCP #		1		2			3				4					
Total Bilirubin (mg/dL)	6.6	6.2	6.4	8.6	9.2	–	8.4	4.2	3.3	6.9	9.8	3.2	2.9	2.7	2.4	0.6
Direct Bilirubin (mg/dL)	4.5	4.3	–	5.8	5.8	–	–	1.70	1.6	4.8	5.7	–	1.3	–	1.1	–
CA 19-9 (unit/mL)		904									7380					13

## Discussion

CA 19-9 functionality comes from its ability to recognize the sialylated Louis A/B antigen on MUC1 [2]. MUC1 is a mucin from a family of multifunctional glycoproteins that is present in the epithelium of the gastrointestinal (GI) tract [2]. Its presence is vital to the lubrication and protection of the GI tract [2]. Mucins can be divided into membrane-bound and secreted groups, with MUC1 belonging to the former [2]. This glycoprotein is expressed in the intralobular ducts [2]. MUC1 has been implicated in a variety of cancers, including pancreatic cancer [2]. 

While MUC1 can be expressed by normal pancreatic tissue, overt expression can be seen in the early stages of pancreatic cancer and will increase with the progression of neoplasia [2]. Its elevation proportionally correlates with a poorer prognosis [2]. However, it is also seen in pancreatitis [2]. It is important to note that MUC1 can play a role in either pro- or anti-inflammatory [3]. Regarding proinflammation, MUC1 can create an environment that allows for tumor proliferation and migration [3]. It can promote epithelial-mesenchymal transition (EMT) by activating the NF-KB P65 pathway, allowing for malignancy to occur [3]. 

While CA 19-9's utility in diagnosing malignancy is remarkable, its positive predictive value is dismal at 72.2% [4]. False positives have been noted due to benign etiologies, especially obstructive disease [4]. The elevation of CA 19-9 in benign processes is well documented. The majority of benign elevations are noted in choledocholithiasis and acute cholangitis [5]. However, the mechanism is not well understood [5]. Proposed mechanisms include increased biliary pressure and subsequent irritation of cholangiocytes or decreased hepatic clearance of mucins secondary to hepatic dysfunction [5]. CA 19-9 should not be used alone in diagnosing malignancy; it is useful along with clinical evaluation and imaging [5]. 

Scara et al. note that in studies that analyze the relationship between CA 19-9 and pancreatic cancer [4]. After extensive analysis, 37 kU/l has been the cutoff limit for CA 19-9 when evaluating pancreatic cancer [4]. However, given the frequency of elevation in benign pathologies, some have suggested using a higher cutoff [6]. With the cutoff of 37 kU/l, the mean sensitivity and mean specificity were found to be 79% and 82%, respectively, as was seen in a systematic review done by Goonetilleke et al. [7]. It has been proposed to increase the cutoff to 100 kU/l or even 1000 kU/l; however, that would inversely increase the specificity and decrease the sensitivity [4,6]. Although raising the cutoff is helpful in the correct clinical context, it is imperative to note that benign pathologies can have levels that can surpass 1000 kU/l. Thus, further supporting the idea that these numbers should not be utilized alone [1]. 

Upon a literature review, it has been seen that multiple cases have shown extreme elevation of CA 19-9 in the setting of benign etiologies. Marcouizos et al. report a case of choledocholithiasis where the CA 19-9 level was elevated to over 99,000 U/mL, which decreased two months after surgery [8]. Ghallab et al. report a case of choledocholithiasis with concurrent cholelithiasis where post-ERCP CA 19-9 was found to be 2,192 U/ml [9]. Akimoto et al. report a case of acute cholelithiasis with a CA 19-9 level of 19,392 U/ml, which normalized after surgery as well [10]. Giron et al. report a CA 19-9 level of 12,838.3 U/mL in acute cholangitis secondary to a benign biliary stricture. Elevations to the extent of these cases are not common and can be disarming to the clinician. However, after interventions such as biliary drainage or surgery, levels typically fall to a normal range [5]. Ultimately, the literature is consistent with what was found by Tsen et al. in that choledocholithiasis and cholangitis are the typical pathologies behind the benign elevation of CA 19-9 [5].

## Conclusions

CA 19-9 is a helpful tumor marker when combined with imaging and clinical judgment. The elevation of CA 19-9 in benign pathologies is becoming more apparent. While useful in diagnosing pancreatic cancer, clinicians should interpret values in the context of the patient’s entire clinical picture.
